# 3D-printed valves to assist noninvasive ventilation procedures during the COVID-19 pandemic: a case study

**DOI:** 10.2217/3dp-2020-0017

**Published:** 2021-02-02

**Authors:** Guilherme Arthur Longhitano, Geovany Candido, Leonardo Mendes Ribeiro Machado, Paulo Inforçatti Neto, Marcelo Fernandes de Oliveira, Pedro Yoshito Noritomi, Francisco Guilherme Mais, Victor Luiz de Paula Souza, Jorge Vicente Lopes da Silva

**Affiliations:** 1 ^1^Laboratory for three-dimensional technologies, Center for Information Technology Renato Archer (CTI), Campinas 13069-901, Brazil; 2 ^2^School of Chemical Engineering, University of Campinas, Campinas 13083-852, Brazil; 3 ^3^Independent Researcher

**Keywords:** 3D printing, continuous positive airways pressure, COVID-19, full-face snorkel mask, medical devices, NIV, non-invasive ventilation, selective laser sintering

## Abstract

**Aim:** To produce valves to be used with full-face snorkeling masks for noninvasive ventilation (NIV) procedure during the coronavirus disease 2019 (COVID-19) pandemic. **Materials & methods:** ISINNOVA’s *Charlotte* valves for full-face snorkeling masks used for NIV procedures were redesigned, produced by selective laser sintering additive manufacturing, and submitted to air leakage tests. **Results:** The final model assembly did not present air leakage during the NIV procedure on human models, minimizing risks of air contamination. **Conclusion:** This study shows the feasibility of using additive manufactured valves with snorkel facial masks to support health systems during COVID-19 and possible future pandemics.

## Graphical abstract

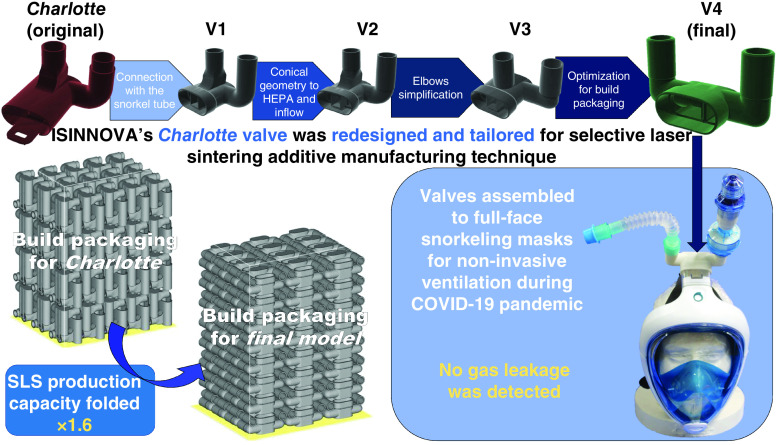

After reaching every continent, the WHO classified the severe acute respiratory syndrome coronavirus-2 (SARS-CoV-2) as a pandemic on 11 March 2020 [1]. Some countries, such as Italy and Spain, presented high infection and death rates but are starting to put in practice reopening plans after significantly lowering the number of new cases [2]. Nevertheless, a recent report from Carlos III public health institute shows that only 5% of the Spanish population was infected with COVID-19 [3]. This represents an alert, showing that if no care is taken during reopening plans, a possible second-wave of contamination may occur [4]. Besides, other countries, mainly from the South hemisphere, are still present growth in the number of cases and deaths [5].

The new coronavirus outbreak generated a high number of patients with severe acute respiratory syndrome (SARS), resulting in a great demand for intensive care unit admission and invasive mechanical ventilation (IMV) procedure [6], also known as intubation [7]. During IMV admission, the health workers are prone to be infected by droplets and aerosols from the patient [8–11]. As an alternative, continuous positive airways pressure (CPAP) and pressure support ventilation (PSV) – noninvasive ventilation (NIV) procedures – can be used as a first approach for continuously providing air and oxygen for patients with SARS. CPAP uses a flow generator and a positive end-expiratory pressure (PEEP) to deliver a continuous positive pressure to the patient and is commonly used for several acute and chronic respiratory diseases, heart failure and sleep apnea treatment [6,7]. PSV, which also uses a PEEP, delivers a continuous airway pressure in a cyclic mode triggered by the patient’s breathing, supporting the whole inspiratory phase [12]. In a study, presented by Wang *et al.* [13], from 27 patients with SARS submitted to noninvasive oxygen therapy, only four were submitted to IMV. If used with a full-face mask device, NIV may decrease virus dissemination through aerosol and droplets, minimizing ambient contamination [6]. Another advantage is that the devices, except for the expiratory filter, can be sterilized and reused [14]. It is worth to mention that they are not competitive technologies since the NIV does not substitute IMV, but can be used as a first well-monitored approach before intubation in patients with lower comorbidities [15,16].

The rapid spread and the high number of patients created a supply chain disruption, originating a lack of resources for fighting off the COVID-19 crisis [17,18]. During the pandemic, initiatives from individuals, groups and organizations in producing devices by 3D printing (additive manufacturing) for helping combat the virus are being massively circulated in media. Various devices, such as face masks, face shields, valves, connectors, test swabs and door handle attachments [17,19,20] gained notoriety. The STL file format, as the standard for additive manufacturing after the mechanical design of such devices, can be easily found on the internet in makers’ webpages such as Thingverse [21]. In Italy, part of a CPAP device was developed by ISINNOVA (Brescia, Italy) using a snorkeling mask (Surface Snorkeling Mask Easybreath^®^ – Decathlon Villeneuve, d'Ascq, France) and 3D printed valves named *Charlotte* and *Dave.* The STL files are free to download at the ISINNOVA’s website, and fused deposition modeling (FDM) is the recommended additive manufacturing technique for fabricating the valves [22].

In this case study, we redesigned the *Charlotte* valve from the ISINNOVA company for selective laser sintering (SLS) process based on the process specificities and maximizing production. The final model was produced and assembled with snorkeling masks and tested during the NIV procedure on human models.

## Materials & methods

### Valves production

The *Charlotte* valve STL files were obtained from ISINNOVA’s website [22] for testing. From the original model, various modifications were done to reduce the fabrication time and improve fitting to the mask. Each modification was approved by an engineering and medical committee involved with COVID-19. The valve design was modeled in Solidworks^®^ 2016 and Rhinoceros 5. Parts allocation for the build volume optimization during valves production was done using Materialise Magics^®^ 22.

The valves were made by SLS technique in a Sinterstation HiQ and a Sinterstation 2000 machines, both from 3D Systems. SLS is a powder bed fusion additive manufacturing technique that uses powder material as feedstock. During the process, a layer of powder material is deposited and selectively sintered by a CO_2_ laser scan. This step is repeated until a 3D part is obtained [23,24]. Because the nonsintered powder acts as support, one major advantage of the SLS is to avoid support structures for parts with complex geometries and the possibility to stack parts during production, increasing productivity. Also, the nonsintered powder can be recycled and reused in the next SLS building process [23,24]. The material used to build valves is Duraform^®^ PA from 3D Systems, a standard Polyamide 12 based powder material for SLS additive manufacturing technique. The processing parameters for virgin powder material for both machines are presented in Table 1.

**Table 1.** Processing parameters for Sinterstation 2000 and Sinterstation HiQ selective laser sintering machines used in valves production.

Parameters	Sinterstation 2000	Sinterstation HiQ
Part heater set point (°C)	180	175
Part cylinder heater set point (°C)	130	130
Left/right feed heater set point (°C)	100	133
Fill laser power (W)	5	15
Outline laser power (W)	–	7.5
Laser scan speed (mm/s)	2000	10,000
Powder layer thickness (mm)	0.1	0.1
Slicer fill scan spacing/hatch spacing (mm)	0.15	0.15
Scale X	1.0446	1.0265
Scale Y	1.0310	1.0264

After production, the parts were removed from the build envelope (partcake) and exceeding nonsintered powder was removed manually. Lose and low-sintered powder from the build envelope were scrapped. The reminiscent powder from the build envelope was mixed with overflow cartridges powder and sifted in a Pilot RBF-15 sieve (VORTI-SIV) with an 80-mesh screen. The obtained powder was recycled for the next builds. To avoid any residual nonsintered powder on the surfaces, the valves were shot-peened using glass microspheres and cleaned with compressed air.

### Masks assembly

**Figure 1.**
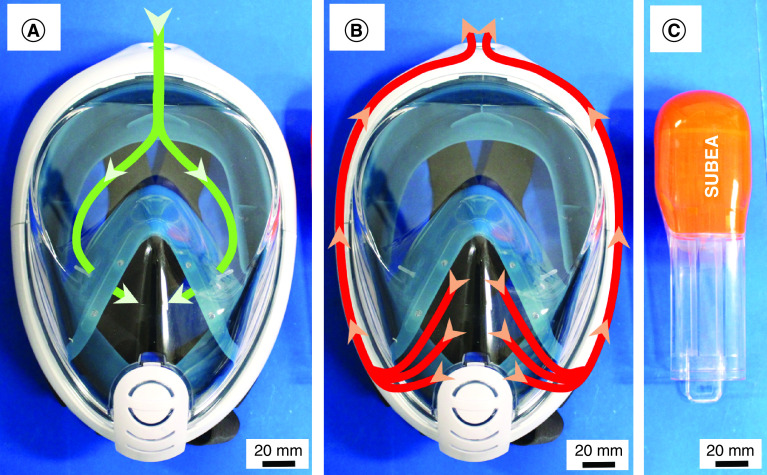
Decathlon’s Surface Snorkeling Mask Easybreath^®^. **(A)** Independent air intake and **(B)** outlet. **(C)** Snorkel tube.

Snorkeling mask sets (Decathlon’s Surface Snorkeling Mask Easybreath) were provided by Decathlon Brazil. The snorkeling mask has independent air channels for inlet and outlet flow (Figure 1). The snorkel tube (Figure 1C) from the set was cut in an automatic band saw and ground for obtaining a smooth surface. The valve and cut snorkel tube were joined using a polyether siloxane sealant glue without solvents and volatile components. All the components (mask, cut snorkel tube with valve, foldable tube, high-efficiency particulate air [HEPA] filter, adaptor tube and PEEP valve) were assembled manually. Polytetrafluoroethylene (PTFE) tape was used to ensure a tight connection between the components.

### Sterilization

The masks and valves were disassembled and washed using a nylon brush and soap. Then, they were put in an ultrasonic cleaner with enzymatic detergent during 20 min. Finally, masks and valves were sterilized by immersion in 0.25% hydrogen peroxide solution for 20 min, dried, reassembled and put in identified plastic bags.

### Air leakage tests

The assembled masks were tested *in situ*, using PSV with a Dräger Savina 300 mechanical ventilator, using volunteer human models from the research team. In this case, the PEEP valve and adaptor tube were not used, and the assembly was linked directly to the mechanical ventilator inlet and outlet. The masks were carefully placed on the model’s head, ensuring a tight fit. The PEEP values were tested between 5 and 12 cmH_2_O. During the tests, the gas volume loss, in %, was obtained from the mechanical ventilator interface screen.

## Results

**Figure 2.**
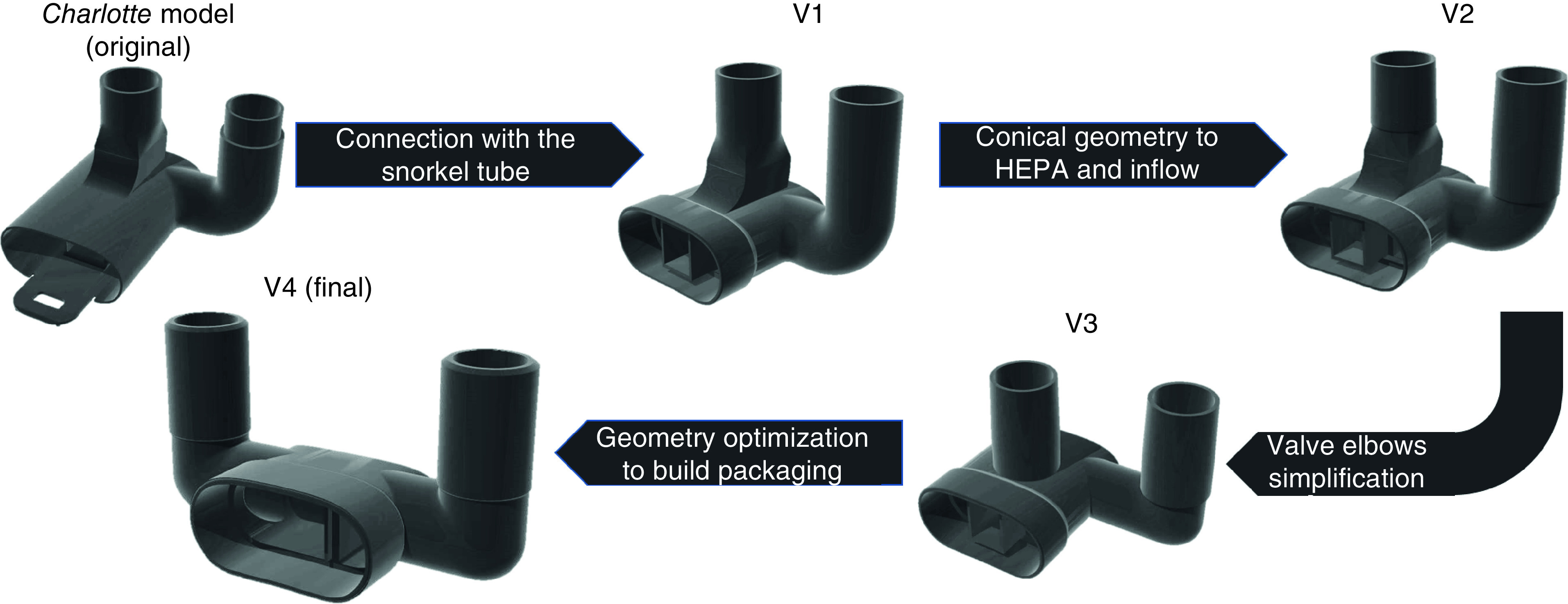
Valves computer-aided design models optimization for the selective laser sintering process. HEPA: High-efficiency particulate air.

**Figure 3.**
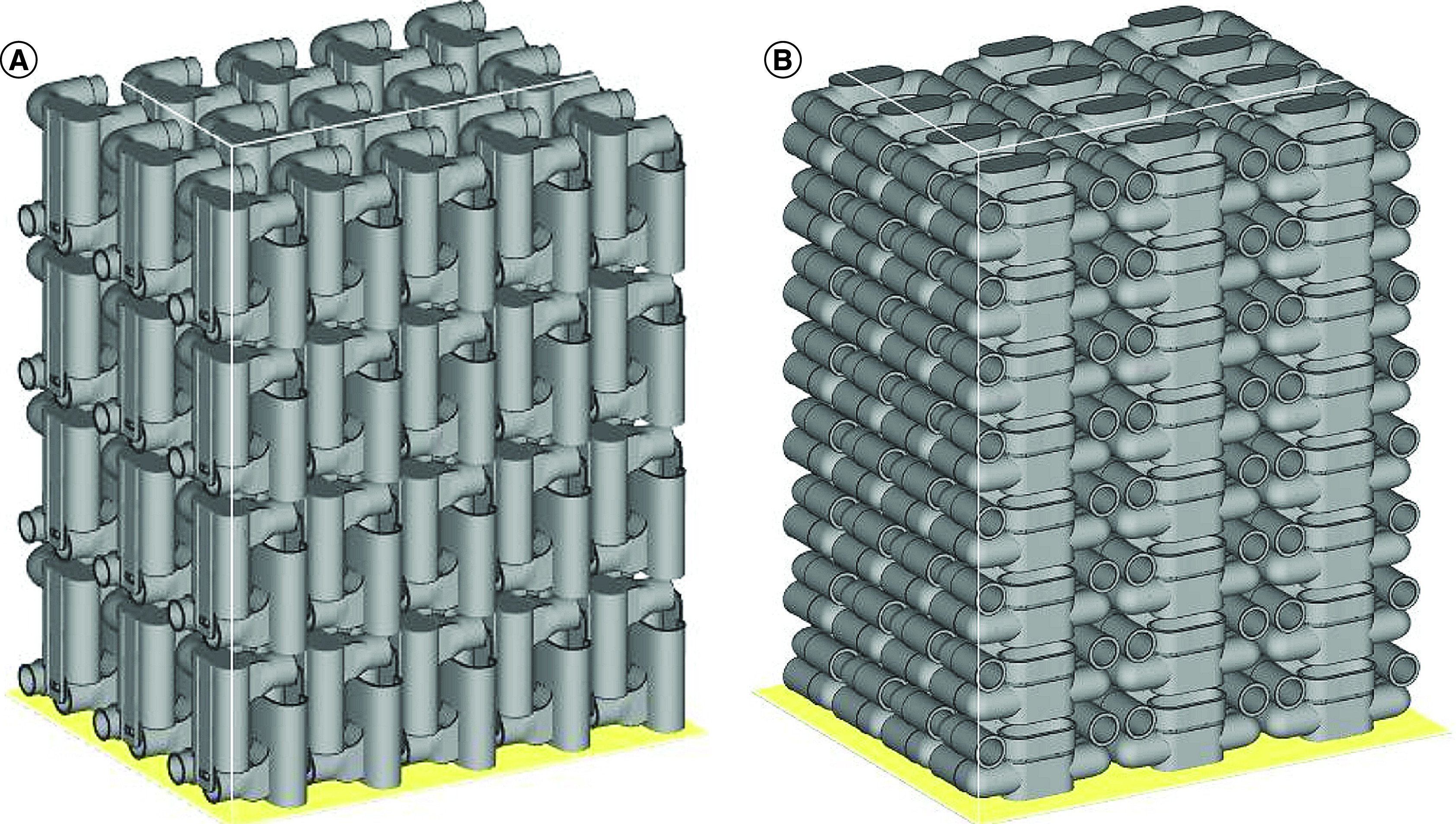
Builds preview in Materialise Magics software for Sinterstation HiQ. **(A)** Original *Charlotte* and **(B)** final version valves build packaging with 120 and 192 pieces, respectively.

In SLS, mechanical properties and geometric tolerances depend on various factors such as powder quality, build orientation and processing parameters. Besides, the process presents high roughness values of surface finish [25]. Therefore, the original *Charlotte* valve project (Figure 2) presented a poor connection and did not ensure a good tightness with the mask in/out opening. To avoid this, diverse valve designs were created and manufactured to minimize any gas leak in the final assembly. In the first model version, the connection channel was changed to be coupled with the snorkel tube from the mask set. In the second version, the tubes connected to the HEPA filter and the foldable tube were modified to a conical geometry to facilitate mechanical connection. The third model simplified the valve elbows. Finally, the fourth and last version was optimized for maximizing the build packaging and optimization, increasing the production capacity. The final model was reviewed by a healthcare professional working with COVID-19 patients. For the original *Charlotte* valve, 120 pieces could be produced per build in Sinterstation HiQ system. The last project increased this number to 192. The parts distribution for build optimization is shown in Figure 3. The final version of the valve can be downloaded on two webpages [26,27]. Both STL and STEP files are given.

**Figure 4.**
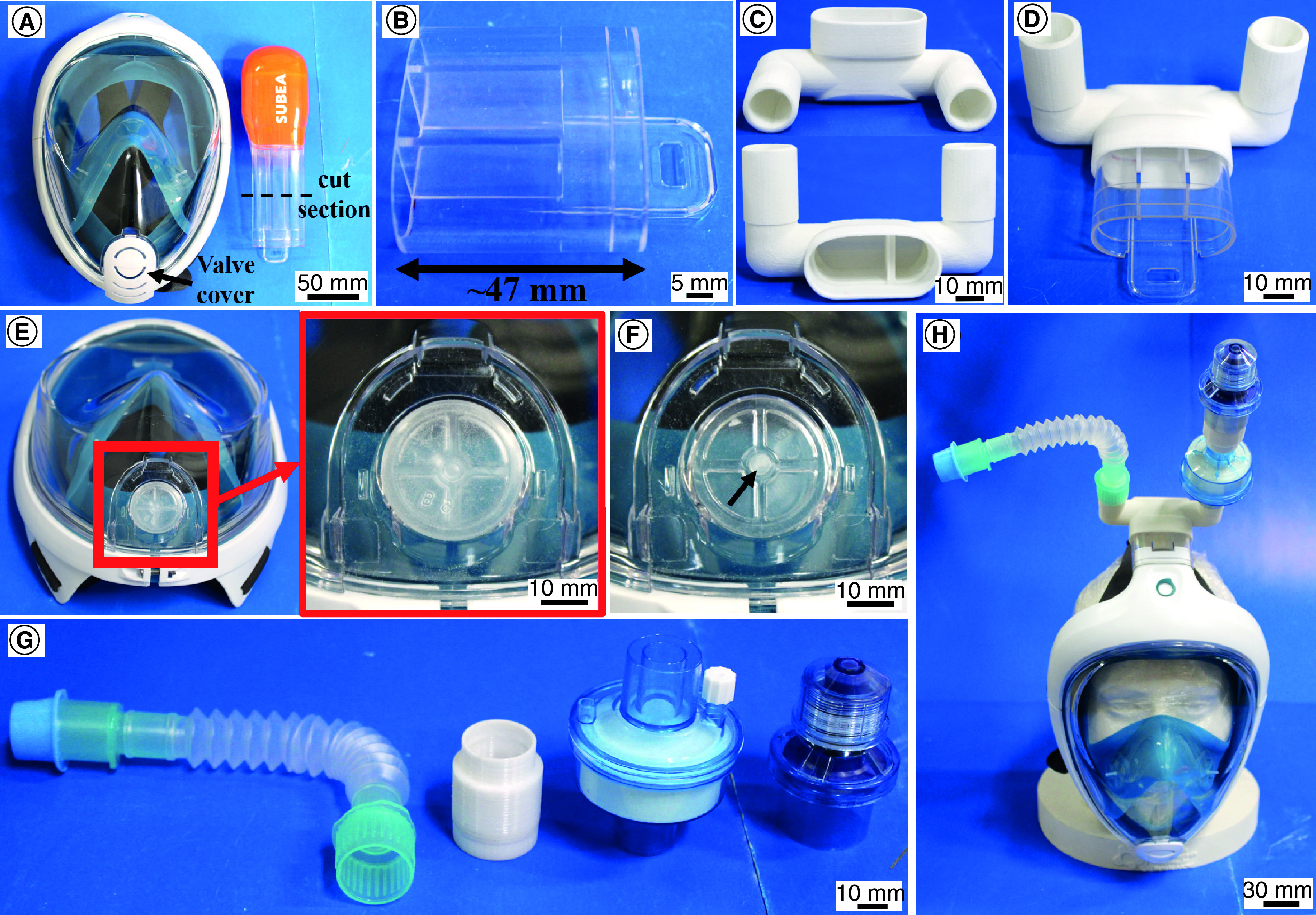
Components and assembly steps for obtaining the mask for continuous positive airways pressure procedure. **(A)** Snorkeling mask. **(B)** Cut snorkel tube. **(C)** Valve produced by selective laser sintering. **(D)** Valve and cut snorkel tube assembly. **(E)** Front valve membrane in normal position. **(F)** Inverted front valve membrane (black arrow shows the silicon valve central pin). **(G)** From left to right: foldable tube, adaptor tube, high-efficiency particulate air filter and positive end-expiratory pressure valve. **(H)** Final mask assembly in a mannequin.

Figure 4 presents all the components and assembly steps. The final valve model was designed to be connected with a cut part of the snorkel tube from the original snorkeling set. The mask presents an O-ring on the in/out opening that ensures a tight connection with the original snorkel tube but has a poor connection with the SLS pieces. Then, the snorkel tube was cut ∼47 mm above the bottom face of the channel (Figure 4B), connected and fixed to the valve using a polyether siloxane sealant glue without solvents and volatile components (Figure 4C & D). The original snorkel mask contains a frontal diaphragm valve (Figure 4E) designed to allow air to exhale and prevent water from entering during dive since the positive pressure is in the outside of the mask. For this application as a NIV support device, therefore, this valve must be blocked or inverted (Figure 4F) since the use as a NIV component has to prevent air from inside the mask from leaking in the ambient because, for this use, the positive pressure is inside the mask. For inverting the valve, the valve cover (Figure 4A) must be removed. The valve is then manually removed and inserted from the inside of the mask, positioning the central silicon pin in the hole (Figure 4F). Then, the valve cover can be reinserted for protection. Finally, a foldable tube and a HEPA filter, in series with an adaptor tube and a PEEP valve (Figure 4G), were linked to the valve inlet and outlet, respectively. The foldable tube is optional since it only has the function to facilitate the air connection. The adaptor tube presents a simple geometry and must ensure the connection between the HEPA filter exit with the PEEP valve inlet. This connector can be easily fabricated by additive manufacturing or injection molding because of its simple geometry. This assembly was designed for use with an oxygen flow/dispenser. If the mask is to be used with a mechanical ventilator, the adaptor tube and the PEEP valve are unnecessary since the equipment controls the inlet and outlet pressures. PTFE tape was used to ensure the tight connections between the components.

Finally, the air leakage tests indicated a 0% gas volume loss during all the 5–12 cmH_2_O PEEP interval for the three tested human models.

## Discussion

In this case study, full-face masks with additive manufactured valves to be used as medical devices in NIV procedure were assembled. During initial tests, four different models were created before achieving the final optimized model. Design for additive manufacturing was considered during all optimization process. For rapid prototyping and a low number of pieces, additive manufacturing is the best choice [28]. The total amount of masks immediately available was 2200 masks donated from Decathlon and consequently represents the number of valves to be produced. In our facilities, we reached a 360 valve production capacity per week. Therefore, to produce all the 2200 valves, a total of 7 weeks was necessary.

When compared with conventional manufacturing techniques, additive manufacturing is advantageous for small batches. However, for big batches, injection molding, for example, is economically favorable [28]. Kunkel *et al.* [29] compared additive manufacturing and injection molding for mass production of face-shields in a preprinted work. The production capacity was 10 parts/day using fused filament fabrication additive manufacturing technique, while injection molding achieved a 2000 parts/day capacity. However, in the present study, if fabricated by injection molding, the parts would require a mold with a side-action cam mechanism, a complex and expensive type of mold with moving parts. In this case, the low number of pieces and complex mold make injection molding choice not feasible. Even that the number of pieces was considered high for additive manufacturing production, the emergency and the availability of machines and material in our laboratory made feasible the SLS process to quickly respond to the crisis.

The *Charlotte* valve model was designed originally for the FDM process. Since this technology is accessible as low-end machines, people who own desktop printers can quickly start printing at home, but there are some risks associated with the ‘domestic production’ of such valves as quality control and repeatability. Therefore, even for the same STL file, 3D printer machines have differences in software, calibration and thermoplastic quality, resulting in parts with heterogeneous quality [30].

Even with a shortage of equipment and resources, it is mandatory that emergency previously not certified medical devices be produced and tested such certain controlled conditions, avoiding any preventable harm to patients and healthcare workers [20]. In this context, it is important to remark the regulatory agencies issue. Even if validated by a medical and engineers team, the providers’ country regulatory agency (such as the US FDA) must approve the medical device for use, which is usually an expensive and time-consuming process [31]. However, during the pandemic, regulatory agencies published declarations on using these devices during the emergency [32,33].

The final valves model was designed based on the assembly tightness and production speed. Since SLS was the chosen technology for manufacturing, it is unnecessary to plan support structures for the parts during the building process, like in techniques such as FDM. Another advantage of the SLS technique is that it allows easy stacking of parts, maximizing build capacity and productivity. By remodeling the valve, the production capacity was 1.6-folded if compared with the original *Charlotte* model. The material was Duraform PA, which is nontoxic, resistant to chemicals, presents low moisture absorption and is compatible with autoclave sterilization [34]. The total amount of time to build parts in SLS can reach 100 h of continuous processing. The Polyamide 12 powder material is recycled after each processing. Each processing can lead to different results, and an experienced user is paramount to adjust processing parameters, ensuring parts quality. Therefore, it is important that different groups involved in additive manufacturing for any emergency could share experience and knowledge and keeping contact with medical professionals to correctly support demands and improvements in the developments.

Brazil is currently the second country with more confirmed cases and deaths [5], and such actions are necessary to contribute to the health system in the country. Furthermore, some authors highlight that the number of cases and deaths in the country are under-reported: the real number of contaminated people might be three- to ten-times higher than reported numbers [35]. Health systems are becoming overloaded and the need for medical supplies and devices is increasing consistently. For severe cases, CPAP is an alternative to IMV procedure, increasing the patients’ chances of survival, and reducing health workers’ risk of contamination [6,8,9]. Besides, since the procedure can be done only with an external oxygen source, a mechanical ventilator is optional. This is an important issue since a mechanical ventilator shortage during the pandemic in the country is expected [36]. In a study made by Pavlov *et al.* [11], it is shown that diverse noninvasive options for providing oxygen or respiratory support are prone to disperse aerosol particles. Venturi masks, which can be used for noninvasive positive pressure ventilation process, presented a maximum exhaled dispersion distance from 33 cm at FiO2 40% to 40 cm at 24%, creating a high contamination risk for healthcare workers. In a review, Winck & Ambrosino [37] point out that NIV used for new coronavirus patients with inappropriate seals should not be used. The mask used in this study was originally projected for snorkeling practice. During snorkeling, water generates a higher pressure outside the mask. Used as a full-face mask for CPAP, the air source generates a higher pressure inside the mask. Nevertheless, the mask assembly did not present any ambient contamination. When used with a mechanical ventilator, the apparatus indicated the mask could hold all the air inside the system without presenting air leakage. Thus, the full-face snorkeling mask assembly minimizes the risk of contamination for people who share the patient’s ambiance. Following these findings, Kroo *et al.* [38] used snorkel masks connected with 3D printed valves as personal protective equipment and found the equipment capable of exceeding the standards of half-face or N95 respirators.

When infected with SARS-CoV-2, patients may present respiratory distress, with respiratory rates over 30 cycles/min and low PaO2/FiO2 levels (<150 mm Hg) [9]. If no action is taken for patients with these symptoms, progressive multi-organ dysfunction may occur, leading to death. During these acute hypoxemic symptoms, patients are generally submitted to IMV [39]. However, in these cases, if the patient is awake, NIV procedure should be the first option since after intubation patients may remain in ICU in an induced coma for long periods, increasing the risk of death from the use of sedatives. The noninvasive positive pressure ventilation processes for COVID-19 patients were recommended by the WHO [16]. During the procedure, the positive pressure improves the gas exchanging surface inside the patient’s lungs (maintaining alveoli open), and a controlled FiO2 also improves oxygen supply. Finally, it is important to remark that this procedure should not be made in obtunded patients since the entry of air into the stomach may lead to gastric insufflation, regurgitation, aspiration and pulmonary damage [40,41].

We hope the present study can support groups worldwide that are on the fierce fight against equipment and supplies shortage during the COVID-19 pandemic crisis. It highlights the importance of additive manufacturing as a technique for immediate response during emergencies.

## Conclusion

During the COVID-19 pandemic, the shortage of equipment and resources made additive manufacturing an immediate-response fabrication method. SLS additive manufacturing technique was used to rapidly manufacture prototypes and a series of 2200 valves for final use and integrated into snorkeling facial masks as part of medical devices to support NIV systems in hospitals. The mask assembly did not present air leakage, minimizing the air contamination around the patient and reducing risks for the healthcare providers. This study showed the feasibility of using additive manufactured valves with snorkel facial masks to support medical devices for NIV procedures. It is worth saying that this emergency device is not yet cleared by the Brazilian National Surveillance Agency (ANVISA), but there is a protocol opened for this purpose.

## Future perspective

During the COVID-19 pandemic, additive manufacturing has been proven as an emerging technology, capable of producing items in shortage or newly designed parts in a short time and in a decentralized way. It could be used for prototype and final-use parts, saving time and lives. All the devices, such as the valves for NIV procedures, if approved by regulatory agencies, will remain as new medical devices that will help patients in need. Much has been learned during these hard times and, with the development of new materials, technologies and applications, additive manufacturing will certainly be an important tool for fighting the next coming emergencies.

**Summary points**

- The selective laser sintering (SLS) technique can produce complex geometries avoiding support structures.
- SLS also allows easy parts stacking during production.
- The *Charlotte* valve from ISINNOVA company was redesigned for SLS.
- A medical committee involved with COVID-19 approved the new design.
- Valves were produced by SLS in polyamide 12.
- Valves were assembled with surface snorkeling masks.
- Productivity for SLS was 1.6-folded, compared with original *Charlotte* design.
- Masks assembly did not present air leakage.
- Results show the assembly is feasible for a noninvasive ventilation procedure.
